# Toward Sustainable Clinical Analysis: Benchmarking Plastic Use in LC–MS Sample Preparation – Exemplified by Ketamine Analogues in Whole Blood

**DOI:** 10.1021/acs.analchem.5c08225

**Published:** 2026-04-13

**Authors:** Line Noreng, Åse Marit Leere Øiestad, Frederik André Hansen, Hanne Røberg-Larsen, Steven Ray Wilson, Elisabeth Leere Øiestad

**Affiliations:** † Section for Chemical Life Sciences, Department of Chemistry, Faculty of Mathematics and Natural Sciences, University of Oslo, 0315 Oslo, Norway; ‡ Hybrid Technology Hub – Centre for Organ on a Chip-Technology, Institute of Basic Medical Sciences, Faculty of Medicine, University of Oslo, 0317 Oslo, Norway; § Section of Forensic Toxicological Analytics, Department of Forensic Sciences, Division of Laboratory Medicine, 155272 Oslo University Hospital, 0424 Oslo, Norway; ∥ Department of Pharmacy, Faculty of Mathematics and Natural Sciences, University of Oslo, 0316 Oslo, Norway; ⊥ Section of Forensic Research, Department of Forensic Sciences, Division of Laboratory Medicine, 155272 Oslo University Hospital, 0424 Oslo, Norway

## Abstract

The aim of this study was to assess and benchmark plastic consumption in sample preparation for forensic analysis, alongside the development of an LC–MS method for ketamine analogues in whole blood, with various sustainability-related scores and parameters examined throughout. Ketamine analogues are emerging psychoactive substances associated with intoxication and fatalities globally. An analytical method was developed for determining ketamine and eight of its analogues in human whole blood. Focus was placed on traditional analytical parameters but also the consumption of plastics and reagents, an overlooked aspect of clinical and forensic chemistry. We evaluated three sample preparation techniques: protein precipitation (PPT), liquid–liquid extraction (LLE), and electromembrane extraction (EME). While PPT and LLE are well-established techniques used in clinical settings, EME (i.e., electrophoresis across an oil membrane) is a less established but highly promising approach. All three sample preparation approaches demonstrated similar performance with recoveries above 85% and matrix effects averaging approximately 100%. The EME approach was subsequently refined using a Box-Behnken-based design of experiments and was validated according to the American Academy of Forensic Sciences guidelines. All validated parameters were within the limits, suggesting that the tunable EME approach is a potentially valuable tool in clinical chemistry. The amount of plastic consumables per 100 samples was calculated as being 511 g for PPT, 864 g for LLE, and 303 g for EME. Correspondingly, the amount of organic solvent consumption per sample was 470, 1570, and 210 μL, respectively. The AGREEprep scores (ranges from 0 to 1, 1 is best) were 0.48 ± 0.04, 0.36 ± 0.04, and 0.55 ± 0.05, respectively. The rounded value of PPT (arguably the most used approach for related samples) is 0.5 kg per 100 samples, and we propose that this number be used as a benchmark for plastic consumption in today’s sample preparation. This may serve as a practical reference when developing and evaluating future sample preparation strategies. For example, employing EME here allowed for a 40% reduction in plastics compared to the benchmark, illustrating a significant improvement. However, the investigated approaches have a significant use of single-use consumables, inviting sample preparation approaches that can reduce the plastic footprint.

## Graphical abstract

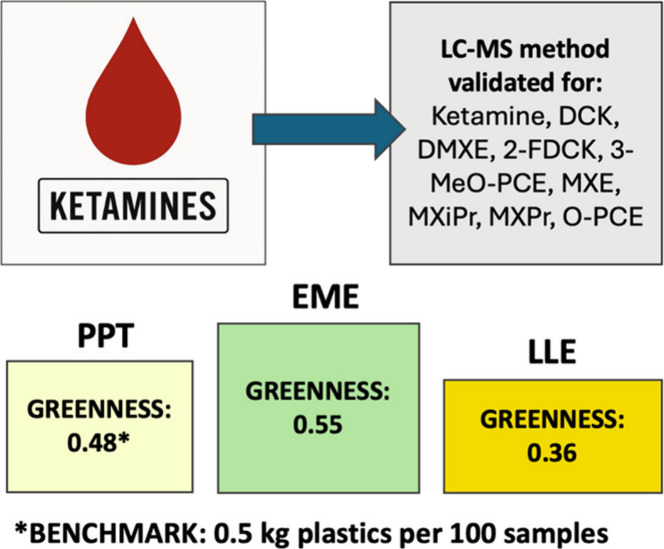

## Introduction

The growing number of detected new psychoactive substances (NPS) in the European drug scene^1^ and globally^2^ requires continuous establishment of analytical methods to support legal regulations, with ketamine analogues (Figure 1A) representing an emerging class of NPS. These drugs are reported to have dissociative and hallucinogenic effects and are associated with both nonfatal and fatal intoxications^3,4^ . Inherent to being analogues, their physicochemical properties are highly similar (Figure 1B), calling for selective measurement platforms. Such platforms typically feature liquid chromatography for analyte separations, electrospray ionization (ESI) for convenient measurement of non-volatile analytes, and novel formats of mass spectrometry (MS) for ultrasensitive and selective measurements. Several methods for a limited number of ketamine analogues have previously been published^5–7^ and some multiplex methods for urine or powder exist^8–11^ , but, to the best of our knowledge, multiplex methods relevant for forensic analysis of whole blood are lacking.

**Figure 1:**
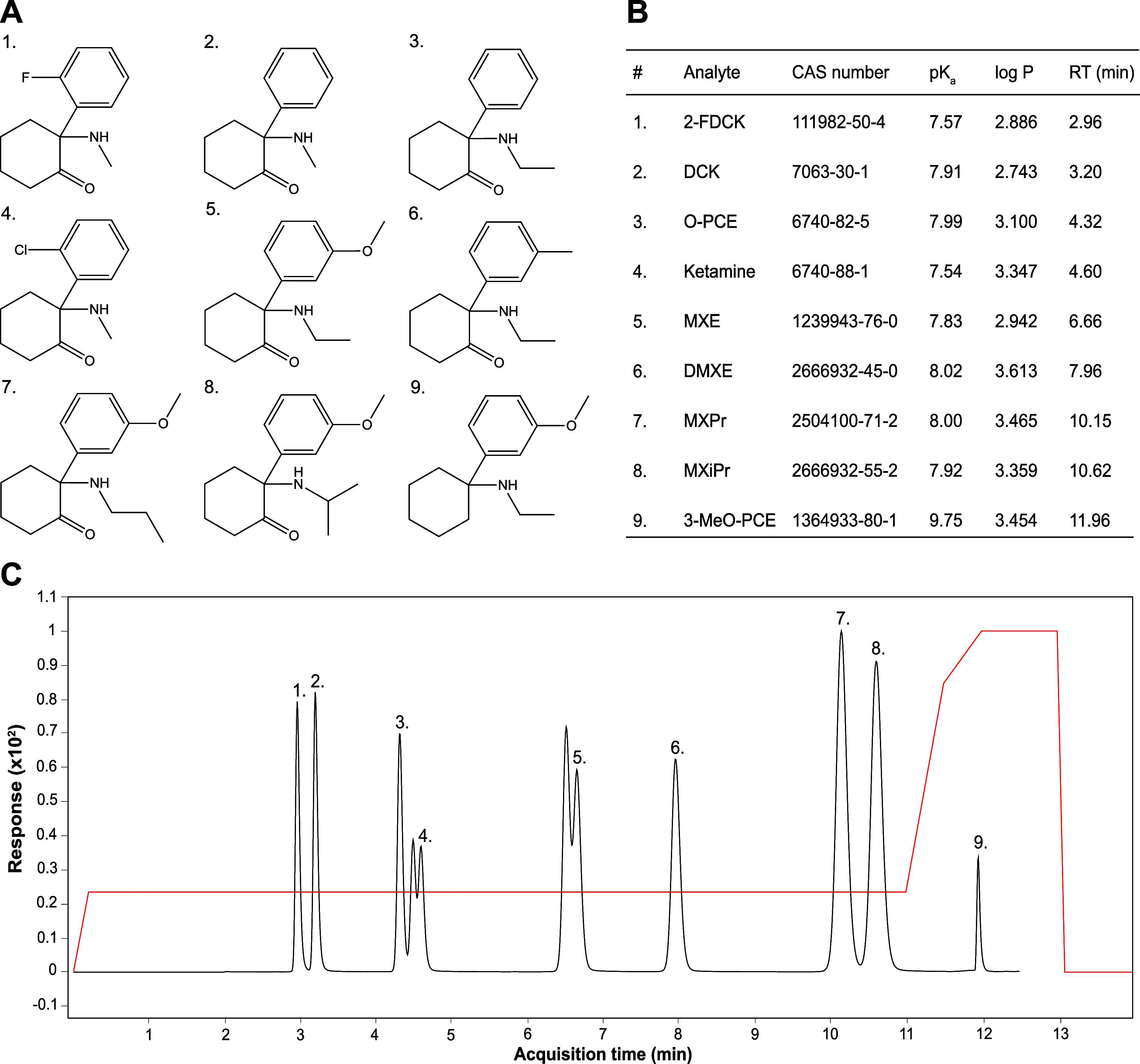
(A) Selected arylcyclohexylamines, commonly referred to as ketamine analogues. Legend: 1, 2-fluorodeschloroketamine (2-FDCK); 2, deschloroketamine (DCK); 3, deschloro-*N*-ethyl-ketamine (O-PCE); 4, ketamine; 5, methoxetamine (MXE); 6, deoxymethoxetamine (DMXE); 7, methoxpropamine (MXPr); 8, methoxisopropamine (MXiPr); and 9, 3-methoxyeticyclidine (3-MeO-PCE). (B) CAS numbers and physicochemical properties (p*K*_a_, log *P*) of ketamine analogues, together with their retention time (RT) in the chromatogram shown in panel (C). (C) Separation of ketamine analogues on a 2.1 mm × 100 mm Kinetex Biphenyl column (1.7 μm, 100 Å, Phenomenex, Torrance, CA, USA) kept at 60 °C with mobile phase (MP) flow rate of 0.6 mL/min (MP A: 10 mM ammonium formate buffer (pH 3.1, adjusted with formic acid); MP B: methanol). The red line indicates the %MP B. Peaks eluting before compounds **4** and **5** are ketamine-d_4_ (used for ISTD correction of 2-FDCK, DCK, O-PCE, and ketamine) and MXE-d_3_ (used for ISTD correction of MXE, DMXE, MXPr, MXiPr, and 3-MeO-PCE), respectively.

Although the abovementioned platforms have seen dramatic advances in the last decades regarding resolution and sensitivity,^12–15^ upstream steps (i.e., the sample preparation) have arguably been more “stand-still” in routine clinical/forensics settings. These workflows still rely largely on classic approaches like protein precipitation (PPT) and (to a lesser degree) liquid–liquid extraction (LLE), although the past decade has seen an increase in the development of microextraction procedures for the determination of NPS in biofluids^16^. Traditional approaches can be prone to considerable manual handling and single use consumables that contribute to the “plastic footprint” of life science (estimated to over 5 million tons per year^17^).

An increased focus on sustainability in chemical analysis is rising (including forensics^18–22^ ), aided by metrics such as the AGREEprep scoring system (0 to 1, with 1 being highest greenness), which provides quantitative frameworks to assess the overall greenness of a sample preparation^23^. However, systematic comparisons and benchmarking of sample preparation greenness in a clinical or forensic context are lacking. We believe an explicit focus is warranted regarding the total plastic spending per method. Motivations include the global threat of plastic pollution^24^, as well as the rapid growth of bioanalysis-related needs and, hence, the number of processed samples.

Our work here has two goals: (a) develop a multiplex method for ketamine analogues suitable for efficient forensic analysis in whole blood to address the increasing number of such compounds used as recreational drugs, and (b) assess and document the greenness of selected sample preparation approaches, as a case study for benchmarking the current state of sustainability in clinical/forensic labs.

Here, we describe comparisons of PPT, LLE, and electromembrane extraction (EME) for measuring ketamine analogues in whole blood. The first two approaches are based on general protocols from our hospital laboratories, which are very likely to resemble many hospital sample preparation protocols. The EME approach has been less explored in clinical settings. EME, essentially an electrophoresis across an oil membrane, provides increased and tunable selectivity.^25–27^ Focus was placed on traditional analytical parameters (recovery, ion suppression, etc.). We also present our comparisons of the methods regarding greenness, using the AGREEprep^23^ scoring system (also evaluating how the scores vary between different users). We then describe our work with optimizing EME and LC–MS settings using design of experiment (DoE) approaches and the validation of the method.

Regarding sample preparation, we present experiments and a critical discussion related to possibilities and bottlenecks for greenness and possibilities that may lie ahead, e.g., miniaturization, automation, and online analysis.

## Materials and Methods

### Chemicals

Type 1 water (18.2 MΩ·cm) was produced using a Milli-Q Advantage A10 system equipped with a Q-POD dispenser and a 0.22 μm filter (Merck KGaA, Darmstadt, Germany). Acetonitrile (ACN, ≥99.9%) was obtained from Avantor Performance Materials (Gliwice, Poland). Ammonia solution (≥32%), ethyl acetate (EtOAc, ≥99.8%), disodium tetraborate decahydrate (≥99%), *n*-heptane (≥99.0%), methanol (MeOH, ≥99.9%), sodium hydroxide (NaOH, ≥99%), and 2-nitrophenyl octyl ether (NPOE, ≥99%) were purchased from Merck KGaA. Ammonium formate (≥98.5%) and formic acid (FA, ≥98%) were obtained from VWR (Leuven, Belgium, and Briare, France, respectively). Nitrogen (N_2_, ≥99.999%) was supplied by AGA (Leirdal, Norway). Human whole blood was obtained from the Oslo University Hospital Blood Bank and stored at –20 °C.

#### Target Analytes (TAs) and Internal Standards (ISTDs)

Ketamine (>98.5%) was obtained from Lipomed AG (Arlesheim, Switzerland). 2-Fluorodeschloroketamine (2-FDCK, 98%) was purchased from Chiron (Trondheim, Norway), while deschloroketamine (DCK, 95%) and methoxetamine (MXE, 98%) were obtained from Toronto Research Chemicals (Toronto, Canada). Deschloro-*N*-ethyl-ketamine (O-PCE, ≥98%), deoxymethoxetamine (DMXE, ≥98%), methoxpropamine (MXPr, ≥98%), methoxisopropamine (MXiPr, ≥98%), and 3-methoxyeticyclidine (3-MeO-PCE, ≥98%), as well as MXE-d_3_ (≥99%) were supplied by Cayman Chemical Company (Ann Arbor, MI, USA). Ketamine-d_4_ (99.4%) was obtained from Cerilliant (Round Rock, TX, USA).

#### Solutions

Stock solutions of TAs and ISTDs were prepared at 150 μM in MeOH/H_2_O (1:1, v/v) and stored at –20 °C. Tuning solutions of each TA and ISTD were prepared at 10 μM in MeOH/H_2_O (1:1, v/v). A TA mixture for ESI and LC optimization was prepared at 1 μM in MeOH/H_2_O (1:1, v/v). Calibration standards and quality control samples containing all TAs were prepared in MeOH/H_2_O (1:1, v/v) in the concentration range of 0.025–10 μM, with five QC samples in the calibration area.

### Tuning MS Parameters and Optimizing ESI Conditions

A 6495D Triple Quadrupole mass spectrometer (Agilent Technologies, Santa Clara, CA, USA) was used. Precursor/product ions and collision energies were identified via the direct infusion of 10 μM TA and ISTD solutions using MassHunter Optimizer (Agilent Technologies). ESI parameters were optimized with a 1 μM TA mixture, starting with sheath gas temperature (200–400 °C), and set finally to 400 °C. Six additional parameters were screened: sheath gas flow, drying gas temperature/flow, nebulizer pressure, capillary voltage, and nozzle voltage. Less significant parameters were set to default values. A Box–Behnken DoE was applied to optimize sheath gas flow (8–12 L/min), capillary voltage (±2500 to ±4500 V), and nozzle voltage (±0 to ±2000 V), resulting in 15 experiments (Table S1). Peak area was used as the response variable, analyzed using Design-Expert (Stat-Ease, Minneapolis, MN, USA). Final optimized conditions were a 12 L/min sheath gas flow, ±2500 V capillary voltage, and ±0 V nozzle voltage.

### Optimizing LC Conditions

A 1290 Infinity II LC system (Agilent Technologies) was used. Three analytical columns were tested: an ACQUITY UPLC BEH C18 (2.1 mm × 100 mm, 1.7 μm, 130 Å) and an ACQUITY UPLC HSS T3 (2.1 mm × 100 mm, 1.8 μm, 100 Å), both from Waters (Wexford, Ireland), and a Kinetex Biphenyl column (2.1 mm × 100 mm, 1.7 μm, 100 Å) from Phenomenex (Torrance, CA, USA). The BEH column was assessed using both acidic (10 mM ammonium formate buffer, pH 3.1, adjusted with FA) and basic (5 mM ammonium formate buffer, pH 10.2, adjusted with ammonium hydroxide) mobile phases, using ACN or MeOH as organic modifiers. The HSS T3 and biphenyl columns were assessed using the same organic modifiers, but only with the acidic buffer. Baseline separation (*R* > 1.5) of the isomer analogues (MXPr and MXiPr) was achieved using the biphenyl column with a temperature of 60 °C and flow rate of 0.6 mL/min (Figure 1C). MP A was 10 mM ammonium formate buffer (pH 3.1, adjusted with FA) and MP B was MeOH with the following gradient: 0 min, 2% MP B; 0.2 min, 25% MP B; 11 min, 25% MP B; 11.5 min, 85% MP B; 12 min, 100% MP B; 13 min, 100% MP B; 13.1 min, 2% MP B; and 14 min, 2% MP B. A divert valve directed flow to the MS from 0.2 to 12.5 min and to waste outside this window. The injection volume was 3 μL.

### Comparing Three Sample Preparation Techniques: PPT, LLE, and EME

The PPT and LLE procedures were based on protocols from Oslo University Hospital, while the EME procedure was based on literature^26,28–30^ , summarized in Table 1. Polystyrene tubes (4.5 mL, 75 mm × 12 mm) were obtained from SARSTEDT AG & Co. KG (Nümbrecht, Germany), low-density polyethylene stoppers (Ø 11–13 mm) from Kartell Spa (Noviglio, Italy), and polypropylene vials (300 μL, 12 mm × 32 mm) with silicone septum caps from Waters Corporation (Milford, MA, USA). Individual and simultaneous vortex was performed with a WIZARD Advanced IR Vortex Mixer (VELP Scientifica, Usmate Velate, Italy) and a VX-2500 Multi-Tube Vortexer (VWR, Radnor, PA, USA), respectively. Centrifugation was done with an Allegra X-15R bench-top centrifuge (Beckman Coulter, Palo Alto, CA, USA). EME was done with a prototype for commercial EME equipment (Extraction Technologies Norway, Ski, Norway), which comprises ten EME cells.^31^ Each EME cell consisted of a 600 μL donor vial connected to a 200 μL acceptor vial by a union holding a polypropylene membrane (Extraction Technologies Norway). Electric field was applied with an EA-PS 3200-02 power supply (Delta Elektronika, Zierikzee, The Netherlands).

**Table 1:** Sample Preparation Protocols for Protein Precipitation (PPT), Liquid–Liquid Extraction (LLE), and Electromembrane Extraction (EME)

PPT	LLE	EME
• Transfer 100 μL of sample into a polystyrene tube	• Transfer 100 μL of sample into a polystyrene tube	• Transfer 100 μL of sample into a 600 μL donor vial and close the vial with a screw cap until sample preparation
• Add 20 μL of ISTD mixture	• Add 20 μL of ISTD mixture	• Remove and discard the screw cap, then add 20 μL of ISTD mixture
• Add 400 μL of ACN/MeOH (85:15, v/v)	• Add 250 μL of borate buffer (pH 11, adjusted with NaOH)	• Add 80 μL of 25 mM FA
• Vortex (individually) for 5 s at 2000 rpm	• Add 1.2 mL of EtOAc/*n*-heptane (4:1, v/v)	• Mount a union with a porous membrane onto the donor vial
• Vortex (simultaneously) for 1 min at 2400 rpm	• Vortex (individually) for 5 s at 2000 rpm	• Soak the membrane with 10 μL of NPOE
• Cool for 10 min at –20 °C	• Vortex (simultaneously) for 1 min at 2400 rpm	• Transfer 100 μL of 50 mM FA into a 200 μL acceptor vial
• Centrifuge for 10 min at 4500 rpm and 4 °C	• Centrifuge for 15 min at 4500 rpm and 4 °C	• Mount the union with the donor vial onto the acceptor vial
• Transfer 50 μL of supernatant to a polypropylene vial	• Transfer 950 μL of supernatant to a new polystyrene tube	• Place the EME cell in the EME device for extraction for 15 min at 70 V
• Add 50 μL of 10 mM ammonium formate buffer (pH 3.1, adjusted with FA)	• Evaporate to dryness for 20 min at 40 °C	• Close the acceptor vial with a new screw cap
• Vortex (individually) for 10 s at 2000 rpm	• Redissolve in 100 μL of 10 mM ammonium formate buffer (pH 3.1, adjusted with FA) with 10% MeOH	**Total number of steps involving electrical appliances:**
**Total number of steps involving electrical appliances:**	• Vortex (simultaneously) for 30 s at 2400 rpm	**1. Extraction**
**1. Vortex**	• Transfer to a polypropylene vial	
**2. Cooling**	**Total number of steps involving electrical appliances:**	
**3. Centrifugation**	**1. Vortex**	
**4. Vortex**	**2. Centrifugation**	
	**3. Evaporation**	
	**4. Vortex**	

Recoveries were evaluated by preparing three samples spiked before the procedure (PRE 1–3) and three spiked after (POST 1–3). For PRE samples, 20 μL of the TA mixture (15.2 μM for PPT; 1.77 μM for LLE; 1.5 μM for EME) and 20 μL of MeOH/H_2_O (1:1, v/v) were added directly to 100 μL of sample, while POST samples received 40 μL of MeOH/H_2_O (1:1, v/v). After the procedure, PRE samples were supplemented with 20 μL of MeOH/H_2_O (1:1, v/v) and 20 μL of a 0.5 μM ISTD mixture, whereas POST samples were spiked with 20 μL of a TA mixture (1.4 μM for PPT; 1.4 μM for LLE; 1.2 μM for EME) and 20 μL of a 0.5 μM ISTD mixture.

Matrix effects were evaluated by preparing three matrix blank solutions (MATRIX BLANK 1–3). For PPT, the solution contained 50 μL of ACN/MeOH (85:15, v/v), 50 μL of 10 mM ammonium formate buffer (pH 3.1, adjusted with FA), 20 μL of a 1.4 μM TA mixture, and 20 μL of a 0.5 μM ISTD mixture. For LLE, the solution consisted of 100 μL of 10 mM ammonium formate buffer (pH 3.1, adjusted with FA) with 10% MeOH, 20 μL of a 1.4 μM TA mixture, and 20 μL of a 0.5 μM ISTD mixture. For EME, the solution included 80 μL of 50 mM FA, 20 μL of a 1.2 μM TA mixture, and 20 μL of a 0.5 μM ISTD mixture.

Recovery and matrix effects were calculated by using ISTD corrected peak areas in eqs 1 and 2, respectively. Ketamine-d_4_ was used for ISTD correction of 2-FCK, DCK, O-OCE, and ketamine, while MXE-d_3_ was used for ISTD correction of MXE, DMXE, MXPr, MXiPr, and 3-MeO-PCE.

1 $$\mathrm{recovery (\%)}=\frac{\mathrm{PRE}}{\mathrm{POST}}\times 100$$

2 $$\mathrm{matrix effects (\%)}=\frac{\mathrm{POST}}{\mathrm{MATRIX BLANK}}\times 100$$

### Optimizing EME Conditions

A Box–Behnken DoE was applied to optimize FA concentration in the acceptor solution (50–200 mM), extraction potential (30–100 V), and extraction time (5–30 min), resulting in 15 experiments (Table S2). Peak area was used as the response variable, and data were analyzed with Design-Expert software (Stat-Ease). Final conditions were a 50 mM FA concentration, 70 V extraction potential, and 15 min extraction time.

## Method Validation

The final method (Table S3) was validated according to the *Standard Practices for Method Validation in Forensic Toxicology* guidelines issued by the American Academy of Forensic Sciences (AAFS).^32^ The calibration model was first established, followed by the evaluation of the limit of detection (LOD), limit of quantification (LOQ), bias, within-run and between-run precision, (instrument) carryover, matrix effects, and dilution integrity.

### Greenness

Sample preparation greenness was assessed with the AGREEprep tool^23^ by three users, who made calculations independent of each other. Means and standard deviations were calculated from the total AGREEprep scores.

### Reusing Extraction Supplies and Membranes from Two EME Formats: Conductive Vial and Stainless Steel 96-Well Plate

Reusing the complete extraction setup including membranes, which would potentially reduce the use of plastic, was tested. Carryover from reused EME membranes was evaluated by preparing four samples spiked before the procedure (PRE 1–4) and repeating the extraction with four solvent blank samples (SOLVENT BLANK 1–4) using the same membranes. PRE 1–4 were prepared according to the validated EME method with slight modifications to the donor and acceptor solution volumes according to the EME format, resulting in a final analyte concentration of 0.5 μM for all samples. After extraction, the membranes were retained, rinsed with type 1 water, and reassembled into clean unions. Without adding NPOE to the membranes, SOLVENT BLANK 1–4 (donor solution consisting of 25 mM FA only) were subjected to repeated extraction. Before analysis, the ISTD mixture was added to all PRE and SOLVENT BLANK samples. Carryover was calculated using ISTD corrected peak areas in eq 3:

3 $$\mathrm{carryover (\%)}=\frac{\mathrm{SOLVENT BLANK}}{\mathrm{PRE}}\times 100$$

## Results and Discussion

### Optimized LC–MS Conditions

Tuned MRM transitions ensured selective detection of ketamine analytes (Figure 2A; except isomeric analytes). The fragmentation patterns observed in MS/MS largely aligned with those documented in the literature^8,33^ . Regarding the ESI conditions (Figure 2B and 2C), higher sheath gas temperatures and flow rates were favored, along with high capillary voltages and low nozzle voltages. Optimized LC parameters ensured satisfactory separation of the isomeric analytes.

**Figure 2:**
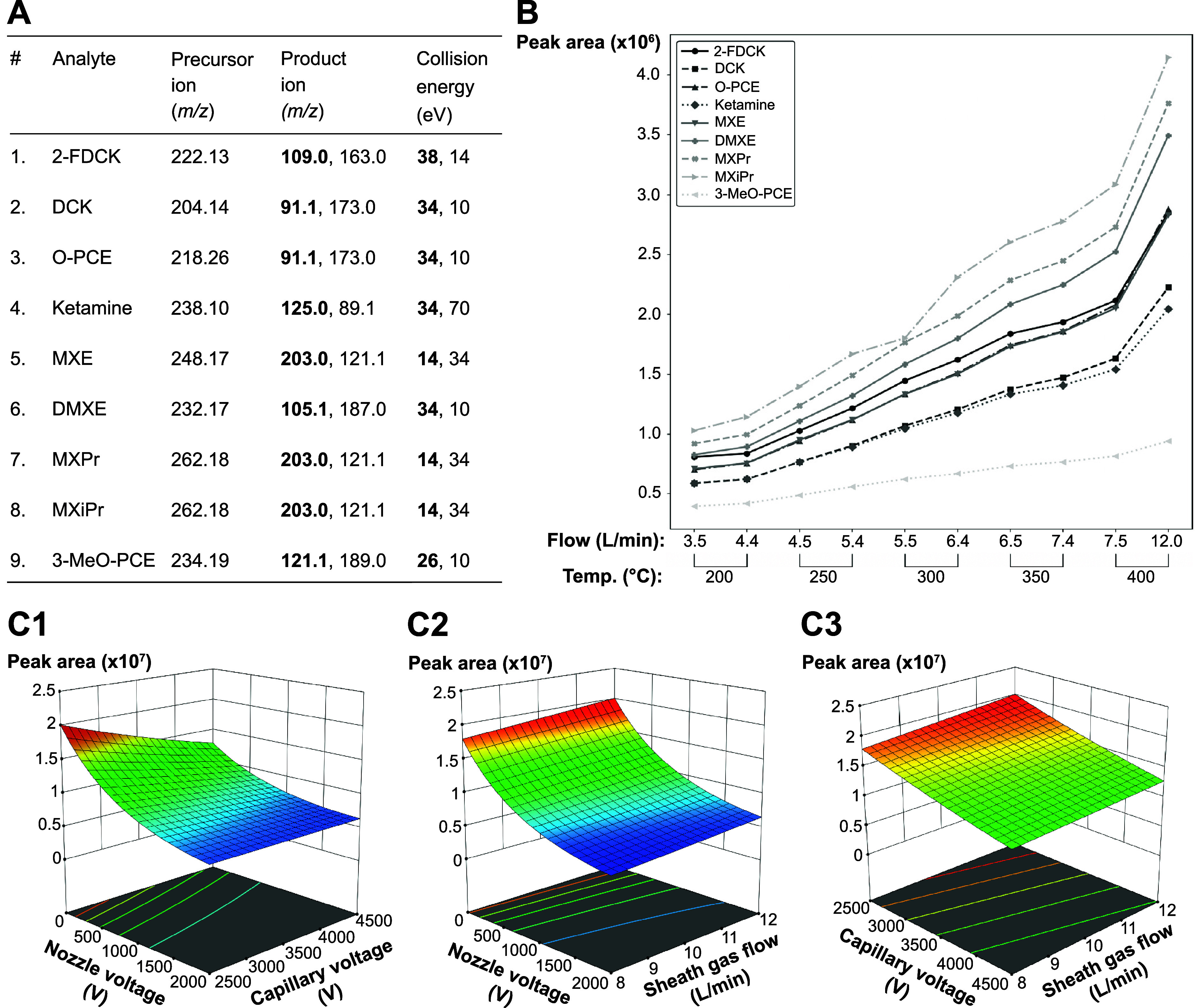
Tuning MS parameters and optimizing ESI conditions. (A) Tuned MRM transitions of ketamine analogues with quantifying (in bold) and qualifying ion. (B) Peak areas of ketamine analogues at sheath gas temperatures ranging from 200 to 400 °C. For each temperature step, flow rates were adjusted to instrumental minimum and maximum limits. (C) Surface plots of ketamine analogue peak area (*z*-axis) as a function of (C1) nozzle and capillary voltage, (C2) nozzle voltage and sheath gas flow, and (C3) capillary voltage and sheath gas flow.

Upon visual inspection of chromatograms, a Kinetex Biphenyl column (2.1 mm × 100 mm, 1.7 μm) combined with MeOH as organic modifier emerged as a suited combination; see Figure 1C for additional details. The isomers MXPr and MXiPr showed a reversed retention order compared with standard C18 behavior, with the straight-chain isomer (MXPr) eluting before the branched-chain isomer (MXiPr). This contrasts with common assumptions in reversed-phase chromatography and is attributed to the specific solvent effects of MeOH: unlike ACN, which can suppress π–π interactions, MeOH facilitates strong π–π interactions and induced polarization between the analytes and the biphenyl stationary phase.^34,35^

### Comparison of Three Sample Preparation Methods: PPT, LLE, and EME

Regarding the sample preparation of the ketamine analogues using PPT, LLE and EME, the recovery and matrix effects were similar (typically >85% and 100%, respectively) (see Figure 3). An exception was the most hydrophobic analyte, 3-MeO-PCE, which showed reduced performance in EME, likely due to its physicochemical properties affecting its electrokinetic migration.

**Figure 3:**
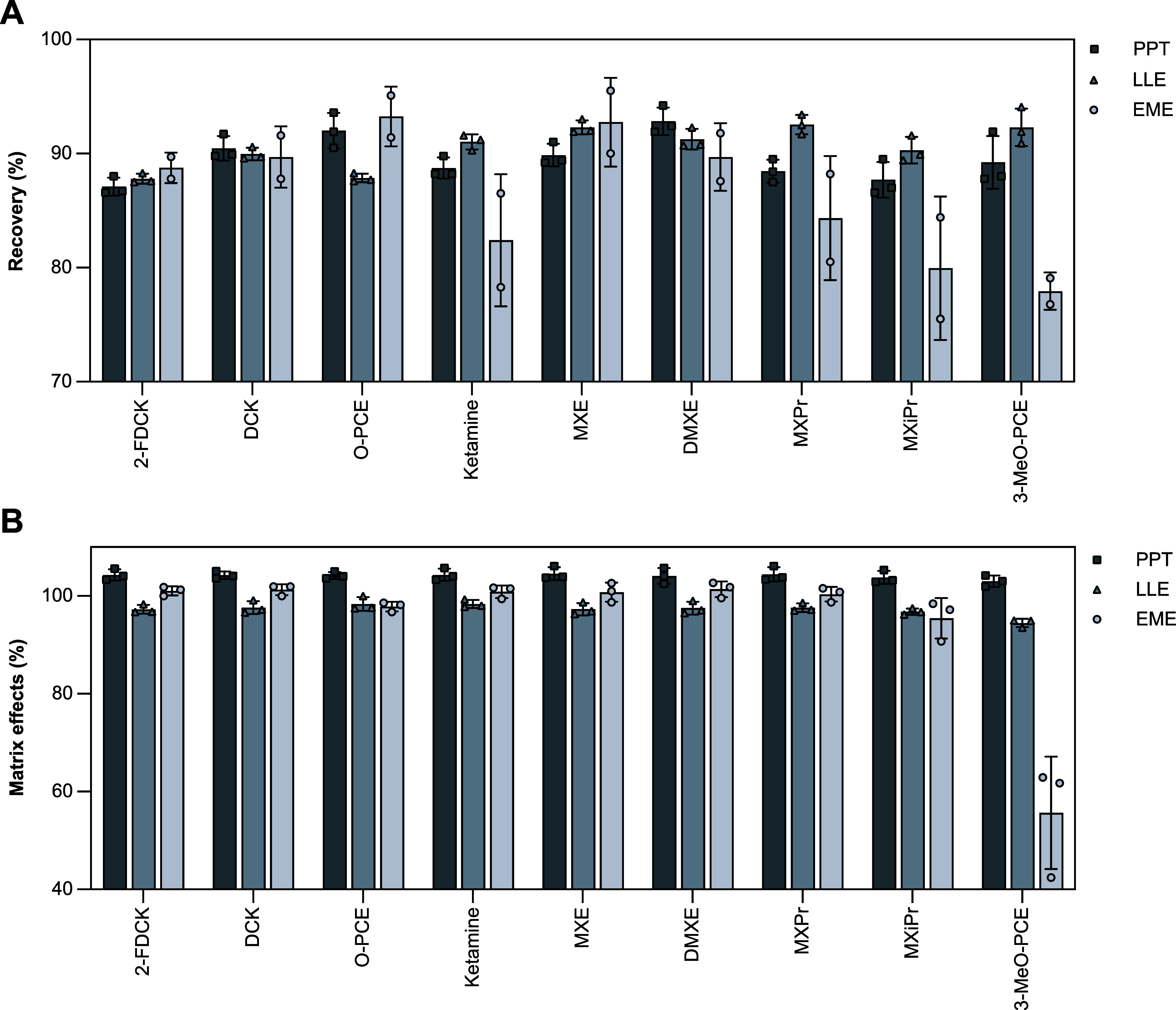
Recovery (A) and matrix effects ((B) with ISTD correction) of ketamine analogues prepared by the described PPT, LLE, and EME methods. The uncorrected matrix effects displayed a similar trend (Figure S1), indicating that all three approaches successfully eliminate major sources of ion suppression and enhancement, even prior to ISTD correction. Standard deviations are included as error bars.

The next step was to map and evaluate the use of consumables and chemicals in the three approaches. A complete list is provided in Table 2. The amount of plastic consumables per 100 samples was calculated as being 511 g for PPT, 864 g for LLE, and 303 g for EME. A more extensive greenness evaluation was undertaken using the AGREEprep scoring system^23^ (where the higher value indicates higher greenness). The scores were, on average, 0.48 ± 0.04 for PPT, 0.36 ± 0.04 for LLE, and 0.55 ± 0.05 for EME (Figure 4). All users evaluated the greenness to be lowest for LLE and highest for EME. The between-user scores varied 8%–11% for all methods, which suggests a decent robustness of the scoring system. However, the results also show that the scoring system can have some subjective interpretation. EME was chosen for further optimization, also due to fewer operator steps (see Table 2), the reduced amount of solvent use (210 μL) vs 470 μL (PPT) and 1570 μL (LLE), and to explore the potential of the less established EME, as it has, for example, possibilities for modifications toward online action (see discussion below).

**Table 2:** Chemicals and Consumables Required per 100 μL of Sample for the Described PPT, LLE, and EME Methods^a^

Technique	Chemicals/100 μL sample	Consumables/100 μL sample
PPT	20 μL ISTD mixture (in MeOH)	2 × pipette tips (200 μL)
	400 μL ACN/MeOH (85:15, v/v)	1 × multipipette tip (2.5 mL)^a^
	50 μL 10 mM ammonium formate buffer (pH 3.1, adjusted with FA)	1 × multipipette tip (5.0 mL)^a^
	**Total: 470 μL**	1 × multipipette tip (50 mL)^a^
		1/25 × multipipette extension^a^
		1 × polypropylene tube
		1 × low-density polypropylene stopper
		1 × polypropylene vial with silicone septum cap
		**Total: 5.11 g (511 g** ^a^ **)**
		
LLE	20 μL ISTD mixture (in MeOH)	2 × pipette tips (200 μL)
	250 μL borate buffer (pH 11, adjusted with NaOH)	1 × pipette tip (1000 μL)
	1.2 mL EtOAc/*n*-heptane (4:1, v/v)	1 × multipipette tip (2.5 mL)^a^
	100 μL 10 mM ammonium formate buffer (pH 3.1, adjusted with FA) with 10 % MeOH	1 × multipipette tip (10 mL)^a^
	**Total: 1570 μL**	1 × multipipette tip (50 mL)^a^
		1/25 × multipipette extension^a^
		2 × polypropylene tubes
		2 × low-density polypropylene stoppers
		1 × polypropylene vial with silicone septum cap
		**Total: 8.64 g (864 g** ^a^ **)**
		
EME	20 μL ISTD mixture (in MeOH)	1 × pipette tip (200 μL)
	80 μL 25 mM FA	1 × multipipette tip (0.1 mL)^a^
	10 μL NPOE	1 × multipipette tip (2.5 mL)^a^
	100 μL 50 mM FA	1 × multipipette tip (10 mL)^a^
	**Total: 210 μL**	1 × donor vial (600 μL)
		1 × polypropylene membrane
		1 × acceptor vial (200 μL)
		2 × screw caps
		**Total: 3.03 g (303 g** ^a^ **)**

a EME unions are regarded as reusable components and are therefore not included among consumables.

b Per 100 samples.

**Figure 4:**
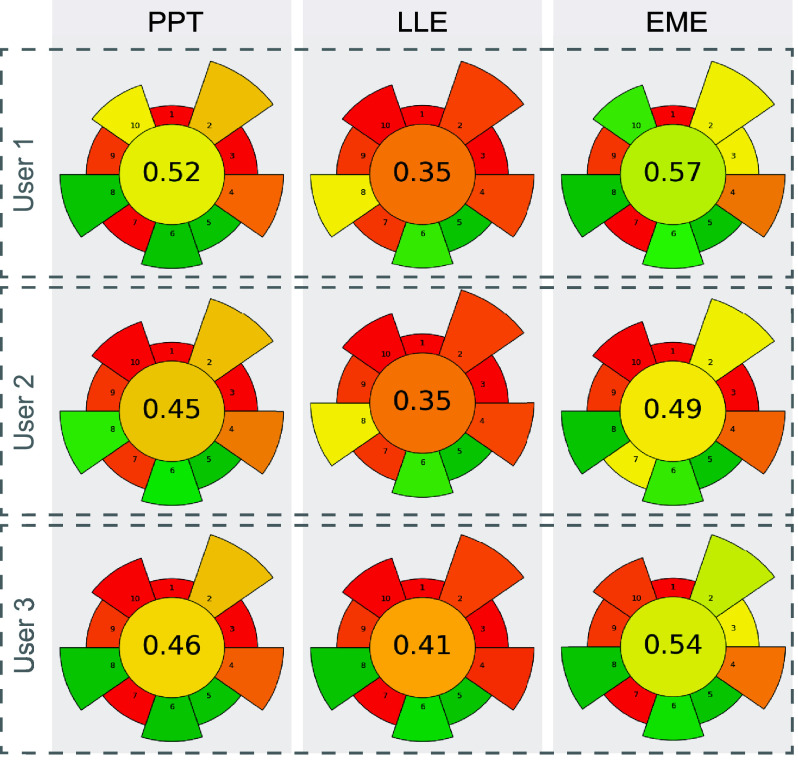
AGREEprep scores of the described PPT, LLE, and EME methods evaluated by three users.

### Optimized EME Conditions

The Box-Behnken DoE of EME parameters (Figure 5A) revealed that the FA concentration in the acceptor solution had a negligible influence, a higher extraction potential was slightly favored, and longer extraction times were highly preferable. Following a practical laboratory approach aimed at increasing sample throughput (i.e., reducing extraction time at the expense of lower recovery), an extraction potential of 70 V across the NPOE oil, an extraction time of 15 minutes, and an FA concentration of 50 mM in the acceptor solution were selected. The optimization efforts allowed the EME approach to mitigate the previous underperformance of the 3-MeO-PCE extraction, increasing recovery from 78% to 102% (Figure 5B) and improving matrix effects from 58% to 72% (Figure S1).

**Figure 5:**
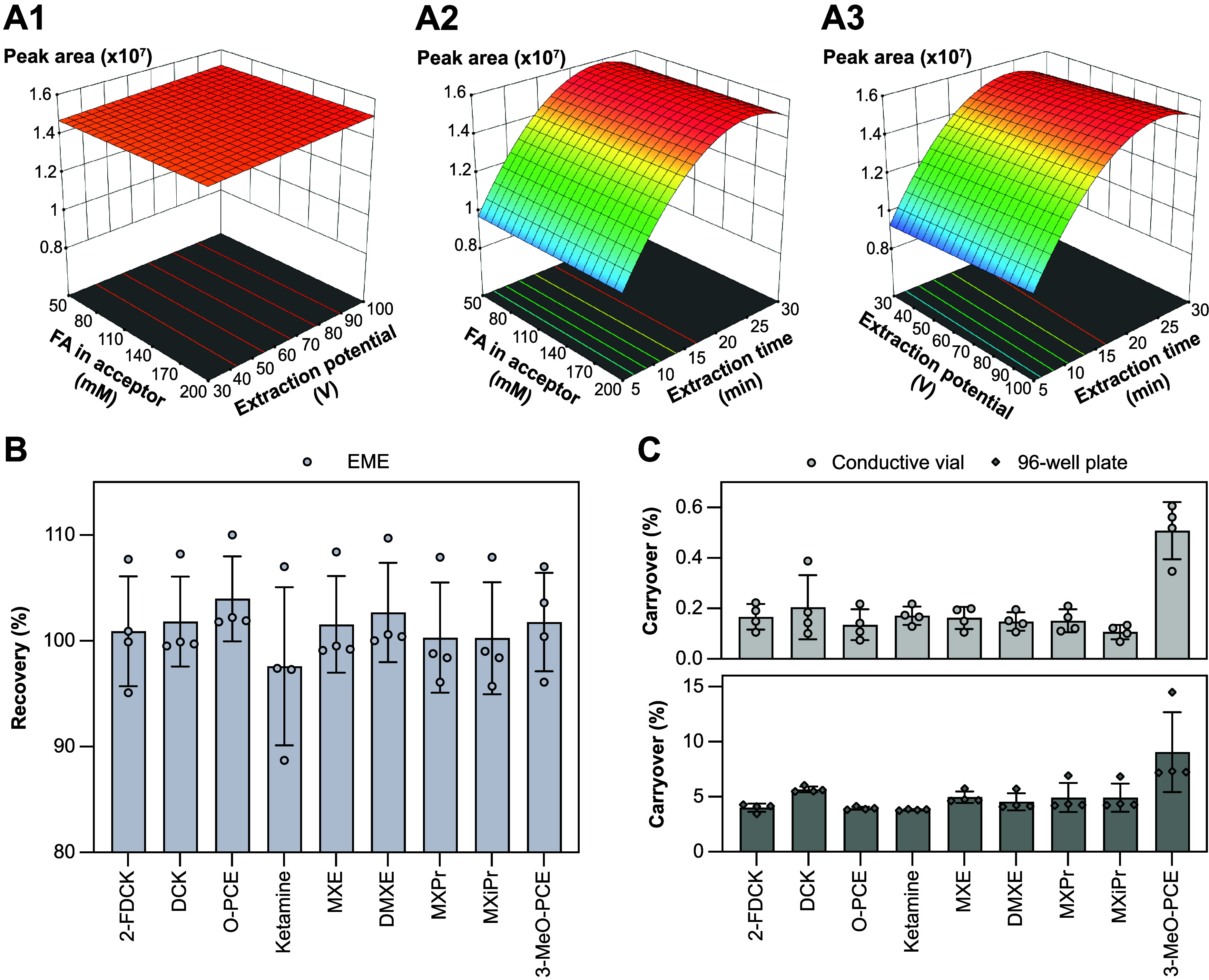
Optimizing EME conditions and reusing extraction supplies and membranes. (A) Surface plots of ketamine analogue peak area (*z*-axis) as a function of (A1) FA concentration in acceptor and extraction potential, (A2) FA concentration in acceptor and extraction time, and (A3) extraction potential and time. The curvature between 15–30 min could result from the model’s poor representation of this region, where the response likely plateaus. (B) Recovery of ketamine analogues prepared by the new EME method. (C) Carryover of ketamine analogues in blank samples after reusing the extraction supplies and membranes from two EME formats: conductive vial and stainless steel 96-well plate.

### Method Validation

The optimized EME–LC–MS method (Table S3) was validated (Tables S4–S8). Calibration curves were linear in the range of 10–2000 nM, corresponding to whole blood concentrations of approximately 2–500 ng/mL. This spans the expected concentrations of ketamine analogues in blood, such as those reported for 2-FDCK (5–480 ng/mL^7^), DCK (40 ng/mL^36^), O-PCE (80–310 ng/mL^6^), MXE (3.6 ng/mL,^5^ 10 ng/mL,^37^ 320 ng/mL^38^), and 3-MeO-PCE (90 ng/mL^39^). To assess higher concentrations, e.g., in overdose scenarios, the method was validated for a 10-fold-sample dilution (Table S7). For instance, Mestria et al. reported high concentrations of 2-FDCK (1300 ng/mL) and MXPr (6400 ng/mL) in a fatality requiring a 1:20 dilution^36^, and Gicquel et al. detected 1780 ng/mL of 2-FDCK in post-mortem peripheral blood.^39^ While the method can be used to detect high concentrations, it must also be sensitive enough to detect impairment at low concentrations, such as driving under the influence. Although regulatory limits for ketamine analogues are not yet explicitly defined in Norway, the legal *per se* limit for ketamine (0.400 μM,^40,41^ ∼95 ng/mL) serves as a relevant reference value. The method achieved LODs of 0.5 nM (0.1 ng/mL) and LOQs of 10 nM (2.0–2.6 ng/mL), well below this threshold. These limits were achieved with an injection volume of 3 μL, corresponding to mass-on-columns of 0.3–0.4 pg (LOD) and 6.1–7.8 pg (LOQ).

The within- and between-day precisions were 0.9%–15% and 0.5%–9.9%, respectively, for all analytes except for 3-MeO-PCE (within 20% for all levels except for within-run measurements at 75 nM, where a CV of 37% was found); the matrix effects were 95%–103% for all analytes, except for 3-MeO-PCE (72%). To assess carryover in potential overdose situations, a very high concentration (20 000 nM; 4–5 mg/mL) was evaluated, resulting in peaks in the following blank sample ranging from 13% to 18% of the lowest calibration standard for all analytes, except for 3-MeO-PCE (29%). Given the AAFS requirement of a maximum carryover of 10%, these results indicate that caution is warranted for samples with very high concentrations, although the European Medicines Agency allows up to 20%,^42^ under which all analytes except 3-MeO-PCE would comply. For samples within the defined range of the calibration curve (10-fold lower concentrations), carryover would fall within both the AAFS and the European Medicines Agency acceptance criteria. Taken together, the EME approach, which has been the subject of limited evaluations in clinical laboratories, was suited for quantitative analysis of ketamine analogues.

We find that ketamine analogues could be successfully measured using EME sample preparation and LC–MS-based separation and analysis, meeting validation criteria. EME is an evolving sample preparation approach, allowing exhaustive removal of interfering proteins^43^, and features fewer operator steps (see Table 2).

### Re-use of Extraction Supplies

Can parts be reused, such as in the case with LC? Regarding multi-use of consumables, we find that oil-loaded EME membranes and conductive plastic vials/stainless steel extraction plates could be reused with new samples and fresh acceptor solution, in the sense that carryover could be acceptable (Figure 5C). This approach was less successful when using a 96-well format EME, likely due to larger observed void volumes than in the vial format. Reuse of EME vials can be useful for nonclinical research but is less of an option in clinical/forensics due to, for example, biohazards and judicial concerns. However, considering that electroextractions are suited for online action^44^ and excellent selectivity, we are currently exploring possibilities for chip-based electroextraction devices to reduce consumables and increase automation, as is the case for the LC column.

### Plastic contaminations

However, in addition to above-discussed environmental issues, plastics can contaminate samples (e.g., leaching), as recently systematically documented for lipidomics.^45^ When performing analysis in untargeted mode (ToF-MS), we also observed similar issues (Figure 6). Such contaminations may contribute to (random) matrix effects.

**Figure 6:**
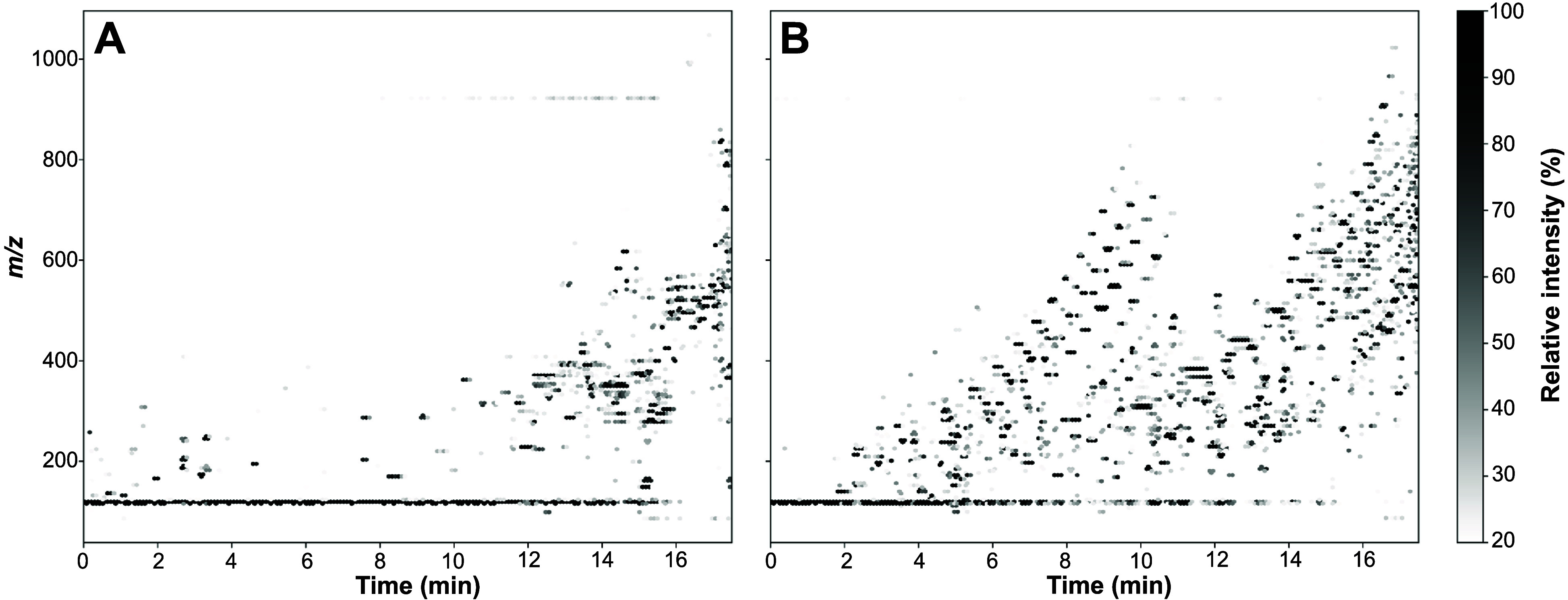
Heatmap (2D) of *m/z* versus retention time of two samples from the study. Curved, repeating units with mass differences of 44 Da can be observed (especially in sample example B), typically associated with polymers. Cutoff is 20% relative intensity.

## Conclusions

The rounded value of PPT (arguably the most common approach) is 0.5 kg per 100 samples; we propose that this number be used as a benchmark for plastic consumption in today’s sample preparation. This may serve as a practical reference point when developing and evaluating future sample preparation strategies.

Comparing with PPT and LLE, we find that EME had a reduced number of steps and clear reductions in single-use plastics (40% reduction of the benchmark), organic solvent, and the highest AGREEprep score of the three tested approaches. The EME approach enabled a satisfactory validation for ketamine analogues in whole blood using LC-MS. EME is further characterized by offering superior selectivity and excellent cleanup of matrix components such as proteins, lipids and phospholipids, salts, and many endogenous metabolites. Future EME development should, however, focus on identifying solvent chemistries that allow for simultaneous extraction of a broader range of analyte hydrophobicity, thereby reducing the need for multiple methods (and thus plastic use) for coverage of analytes with diverse properties. In addition, the application of EME for online extraction appears promising considering the above-mentioned advantages.

In addition to considerable sustainability issues, we (and others) find that plastics can be a source of contamination and interference, apparent when performing full scan analyses of the samples.

We believe our findings support a need for reduction of plastics, online variants of sample preparation, and alternative materials to address sustainability and sensitivity issues related to plastics.

## Supplementary Material



## Notes

Section S1: Optimizing ESI conditions by Box–Behnken DoE (Table S1); Section S2: Optimizing EME conditions by Box–Behnken DoE (Table S2); Section S3: Optimized EME–LC–MS method (Table S3); Section S4: Matrix effects (Figure S1); Section S5: Method validation (Tables S4–S8) (PDF)
