# Supplementary material for: Toward Sustainable Clinical Analysis: Benchmarking Plastic Use in LC–MS Sample Preparation – Exemplified by Ketamine Analogues in Whole Blood
Source: Anal Chem. 2026 Apr 13;98(16):11899–909. doi: 10.1021/acs.analchem.5c08225 (PMC13130169; doi:10.1021/acs.analchem.5c08225)
Supplement: Supplementary file 1 [file ac5c08225_si_001.pdf]

Supporting Information

Toward Sustainable Clinical Analysis: Benchmarking Plastic Use in LC–MS Sample Preparation – Exemplified by Ketamine Analogues in Whole Blood

Line Noreng[1,2], Åse Marit Leere Øiestad[3], Frederik André Hansen[4], Hanne Røberg-Larsen[1,2], Steven Ray Wilson[1,2]*, Elisabeth Leere Øiestad[4,5]

[1]Section for Chemical Life Sciences, Department of Chemistry, Faculty of Mathematics and Natural Sciences, University of Oslo, 0315 Oslo, Norway

[2]Hybrid Technology Hub – Centre for Organ on a Chip-Technology, Institute of Basic Medical Sciences, Faculty of Medicine, University of Oslo, 0317 Oslo, Norway

[3]Section of Forensic Toxicological Analytics, Department of Forensic Sciences, Division of Laboratory Medicine, Oslo University Hospital, 0424 Oslo, Norway

[4]Department of Pharmacy, Faculty of Mathematics and Natural Sciences, University of Oslo, 0316 Oslo, Norway

[5]Section of Forensic Research, Department of Forensic Sciences, Division of Laboratory Medicine, Oslo University Hospital, 0424 Oslo, Norway

* **Corresponding author**. Section for Chemical Life Sciences, Department of Chemistry, Faculty of Mathematics and Natural Sciences, University of Oslo, P.O. Box 1033 Blindern, 0315 Oslo, Norway. E-mail: s.r.h.wilson@kjemi.uio.no (Steven Ray Wilson)

Table of Contents

Supporting Information 1: Optimizing ESI conditions by Box–Behnken DoE S-2

Table S1 .... S-2

Supporting Information 2: Optimizing EME conditions by Box–Behnken DoE S-3

Table S2 .... S-3

Supporting Information 3: Optimized EME–LC–MS method S-4

Table S3 .... S-4

Supporting Information 4: Matrix effects S-5

Figure S1 .... S-5

Supporting Information 5: Method validation S-6

Table S4 .... S-6

Table S5 .... S-6

Table S6 .... S-6

Table S7 .... S-6

Table S8 .... S-7

Supporting Information 1: Optimizing ESI conditions by Box–Behnken DoE

Table S1. Experimental setup of the Box–Behnken design for optimization of three ESI parameters: sheath gas flow, capillary voltage, and nozzle voltage. The response is the average peak area of all ketamine analogue analytes.

Experiment	Order	A: Sheath gas flow		B: Capillary voltage		C: Nozzle voltage		Response:
		Level	Value (L/min)	Level	Value (V)	Level	Value (V)	Peak area
1	12	0	10	0	3500	0	1000	8193500.3
2	7	0	10	0	3500	0	1000	8176695.2
3	6	0	10	0	3500	0	1000	8016873.9
4	1	0	10	-1	2500	-1	0	19837526.1
5	5	-1	8	-1	2500	0	1000	7511280.1
6	8	1	12	-1	2500	0	1000	9350278.5
7	3	0	10	-1	2500	1	2000	5824020.5
8	14	-1	8	0	3500	-1	0	14321976.7
9	11	1	12	0	3500	-1	0	15709571.5
10	15	-1	8	0	3500	1	2000	4942124.8
11	10	1	12	0	3500	1	2000	6383824.8
12	9	0	10	1	4500	-1	0	11912495.5
13	2	-1	8	1	4500	0	1000	6323367.2
14	4	1	12	1	4500	0	1000	7840776.9
15	13	0	10	1	4500	1	2000	5372485.9

Supporting Information 2: Optimizing EME conditions by Box–Behnken DoE

Table S2. Experimental setup of the Box–Behnken design for optimization of three EME parameters: concentration of formic acid (FA) in the acceptor solution, extraction potential, and extraction time. The response is the average peak area of all ketamine analogue analytes.

Experiment	Order	A: FA in acceptor		B: Extraction potential		C: Extraction time		Response:
		Level	Value (mM)	Level	Value (V)	Level	Value (min)	Peak area
1	6	0	100	0	50	0	15	14547175.3
2	8	0	100	0	50	0	15	13918261.1
3	15	0	100	0	50	0	15	14434043.6
4	14	-1	50	-1	30	0	15	14081543.4
5	3	0	100	-1	30	-1	5	8923736.1
6	5	0	100	-1	30	1	30	14248094.1
7	4	1	200	-1	30	0	15	13979214.1
8	10	-1	50	0	50	-1	5	9711069.9
9	7	-1	50	0	50	1	30	14569448.7
10	9	1	200	0	50	-1	5	9673581.1
11	12	1	200	0	50	1	30	15218082.0
12	2	-1	50	1	100	0	15	13888914.2
13	1	0	100	1	100	-1	5	9519395.2
14	11	0	100	1	100	1	30	15505041.3
15	13	1	200	1	100	0	15	14626049.3

Supporting Information 3: Optimized EME–LC–MS method

Table S3. Summary of the optimized analytical method developed for the quantification of ketamine analogues in whole blood, including EME, LC, ESI, and MS parameters.

EME parameters	
Sample volume	100 µL
Donor vial type	600 µL
Concentration of FA in donor	25 mM
Volume of FA in donor	80 µL
Acceptor vial type	200 µL
Concentration of FA in acceptor	50 mM
Volume of FA in acceptor	100 µL
SLM type	NPOE
SLM volume	10 µL
Extraction potential	70 V
Extraction time	15 min
LC parameters	
Injection volume	3 µL
Column	Kinetex™ Biphenyl (2.1×100 mm, 1.7 µm)
Column oven temperature	60 °C
MP A	10 mM ammonium formate buffer pH 3.1
MP B	MeOH
Flow rate	0.6 mL/min
Gradient<br><br><br>*A divert valve directed the LC flow into the MS from 0.2–12.5 min of the gradient and waste before and after.*	0 min; 2% MP B,<br>0.2 min; 25 % MP B,<br>11 min; 25% MP B,<br>11.5 min; 85% MP B,<br>12 min; 100% MP B,<br>13 min; 100% MP B,<br>13.1 min; 2% MP B,<br>14 min; 2% MP B
ESI parameters	
Sheath gas temperature	400 °C
Sheath gas flow	12 L/min
Drying gas temperature	200 °C
Drying gas flow	14 L/min
Nebulizer pressure	20 psi
Capillary voltage	± 2500 V
Nozzle voltage	± 0 V
MS parameters	
2-FDCK	*m/z* 222.1 → **109.0**/163.0 (**38 eV**/14 eV)
DCK	*m/z* 204.1 → **91.1**/173.0 (**34 eV**/10 eV)
O-PCE	*m/z* 218.2 → **91.1**/173.0 (**34 eV**/10 eV)
Ketamine	*m/z* 238.1 → **125.0**/89.1 (**34 eV**/70 eV)
MXE	*m/z* 248.2 → **203.0**/121.1 (**14 eV**/34 eV)
DMXE	*m/z* 232.2 → **105.1**/187.0 (**34 eV**/10 eV)
MXPr	*m/z* 262.2 → **203.0**/121.1 (**14 eV**/34 eV)
MXiPr	*m/z* 262.2 → **203.0**/121.1 (**14 eV**/34 eV)
3-MeO-PCE	*m/z* 234.2 → **121.1**/189.0 (**26 eV**/10 eV)

Supporting Information 4: Matrix effects

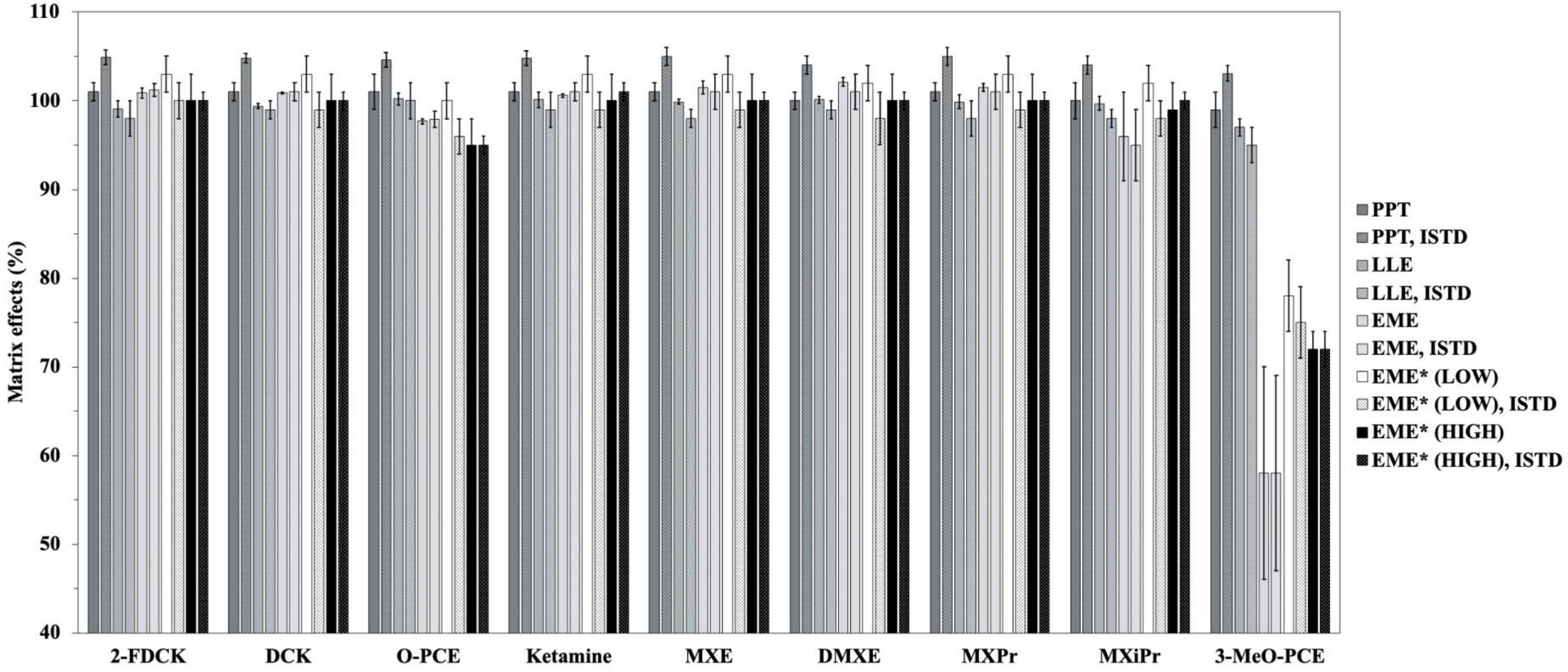

Figure S1. Matrix effects assessed after four sample preparation methods: protein precipitation (PPT), liquid-liquid extraction (LLE), electromembrane extraction (EME), and optimized EME (EME*). Matrix effects after EME* were assessed at low (25 nM; 5.1-6.5 ng/mL) and high (1500 nM; 304.9-392.0 ng/mL) concentration. Matrix effects were calculated without and with internal standard (ISTD) correction for each method and concentration level.

Supporting Information 5: Method validation

Table S4. Calibration model for each ketamine analogue.

Analyte	Calibration range		Model	Weighting	Correlation (R^2)	Origin
	(nM)	(ng/mL)				
2-FDCK	10 – 2000	2.2 – 442.5	Quadratic	1/x	0.9992	Ignore
DCK	10 – 2000	2.0 – 406.6	Quadratic	1/x	0.9997	Ignore
O-PCE	10 – 2000	2.2 – 434.6	Quadratic	1/x	0.9998	Ignore
Ketamine	10 – 2000	2.4 – 475.4	Quadratic	1/x	1.000	Ignore
MXE	10 – 2000	2.5 – 494.7	Quadratic	1/x	1.000	Ignore
DMXE	10 – 2000	2.3 – 462.7	Quadratic	1/x	1.000	Ignore
MXPr	10 – 2000	2.6 – 522.7	Quadratic	1/x	1.000	Ignore
MXiPr	10 – 2000	2.6 – 522.7	Quadratic	1/x	1.000	Ignore
3-MeO-PCE	10 – 2000	2.3 – 466.7	Quadratic	1/x	0.9987	Ignore

Table S5. Limit of detection (LOD) and quantification (LOQ) for each ketamine analogue.

Analyte	LOD			LOQ		
	(nM)	(ng/mL)	(pg on column)*	(nM)	(ng/mL)	(pg on column)*
2-FDCK	0.5	0.1	0.3	10	2.2	6.6
DCK	0.5	0.1	0.3	10	2.0	6.1
O-PCE	0.5	0.1	0.3	10	2.2	6.5
Ketamine	0.5	0.1	0.4	10	2.4	7.1
MXE	0.5	0.1	0.4	10	2.5	7.4
DMXE	0.5	0.1	0.3	10	2.3	6.9
MXPr	0.5	0.1	0.4	10	2.6	7.8
MXiPr	0.5	0.1	0.4	10	2.6	7.8
3-MeO-PCE	0.5	0.1	0.4	10	2.3	7.0

*3 µL injection

Table S6. Carryover assessed by analyzing three blank samples after a standard with very high ketamine analogue concentration (20 000 nM; 4-5 mg/mL; 10-fold the concentration of the highest calibration standard).

Analyte	Blank 1		Blank 2		Blank 3	
	Carryover (%)	% of STD 1	Carryover (%)	% of STD 1	Carryover (%)	% of STD 1
2-FDCK	0.04	13	0.02	6	0.03	7
DCK	0.04	16	0.02	8	0.03	8
O-PCE	0.04	17	0.02	8	0.02	8
Ketamine	0.03	14	0.02	7	0.02	9
MXE	0.03	17	0.02	8	0.02	9
DMXE	0.03	18	0.01	8	0.02	9
MXPr	0.03	18	0.01	8	0.02	10
MXiPr	0.03	18	0.01	8	0.01	9
3-MeO-PCE	0.07	29	0.02	8	0.03	11

Table S7. Dilution integrity assessed at three dilution ratios: 1:1; 1:4; and 1:9.

Analyte	Dilution ratio 1:1		Dilution ratio 1:4		Dilution ratio 1:9	
	Bias (%)	Within-run CV (%)	Bias (%)	Within-run CV (%)	Bias (%)	Within-run CV (%)
2-FDCK	-7.2	3	-12	0.2	-12	5
DCK	-7.3	2	-12	2	-8.4	6
O-PCE	-3.6	2	-8.7	0.6	-3.6	7
Ketamine	-0.23	3	-3.3	0.8	-3.8	3
MXE	-6.1	3	-9.2	1	-10	3
DMXE	-4.5	3	-7.5	1	-9.6	3
MXPr	-3.2	3	-5.4	0.7	-9.1	2
MXiPr	-4.2	3	-6.6	0.7	-11	2
3-MeO-PCE	-2.9	6	-5.5	5	0.96	8

Table S8. Within- and between-run precisions (assessed at five concentrations), bias (assessed at five concentrations) and matrix effects (assessed at one high and one low concentration).

**Analyte**<br>Conc. (nM)	Conc. (ng/mL)	Within-run CV (%)	Between-run CV (%)	Bias (%)	Matrix effects (%)
**2-FDCK**					
1500	331.9	6	4	2.3	99.8
750	166	1*	2	0.65	-
200	44.3	1	1	-11	-
75	17	3	3	-6.2	-
25	5.5	10	6	1.2	103
**DCK**					
1500	304.9	7	5	3.6	100.2
750	152	0.9	2	0.79	-
200	40.7	2	1	-7.7	-
75	15	2	4	-4.5	-
25	5.1	13	9	3.8	103
**O-PCE**					
1500	326,0	7	5	5.2	95
750	163	0.7*	2	2.7	-
200	43,5	3	2	-3.7	-
75	16	3	6	-1.8	-
25	5,4	15	9	4.6	99.8
**Ketamine**					
1500	356.6	3	2	1.3	100.2
750	178	0.6	0.5	1.5	-
200	47.5	0.9	1	3.0	-
75	18	3	2	1.1	-
25	5.9	9	5	1.2	103
**MXE**					
1500	371.0	4	3	0.46	99.8
750	185	1	0.8	1.4	-
200	49.5	1*	2	-3.1	-
75	19	2	2	0.26	-
25	6.2	8	5	0.86	103
**DMXE**					
1500	347.0	4	2	1.6	99.95
750	173	1	0.8	1.7	-
200	46.3	2	2	-1.9	-
75	17	3	2	-0.86	-
25	5.8	9	5	0.43	102
**MXPr**					
1500	392.0	3	2	1.1	99.97
750	196	2*	2	1.4	-
200	52.3	2	2	1.5	-
75	20	3	2	0.059	-
25	6.5	9	5	-0.67	103
**MXiPr**					
1500	392.0	4	3	1.0	99.4
750	196	1	2	2.6	-
200	52.3	2	3	-0.42	-
75	20	4	2	-0.041	-
25	6.5	9	6	-0.28	102
**3-MeO-PCE**					
1500	350.0	14	11	4.9	72
750	175	3*	3	3.1	-
200	46.7	20	14	-1.7	-
75	18	37	19	3.0	-
25	5.8	18	12	4.8	78

*n=2
